# Cost-effectiveness of human papillomavirus (HPV) vaccination in Burkina Faso: a modelling study

**DOI:** 10.1186/s12913-023-10283-3

**Published:** 2023-12-01

**Authors:** Joël Arthur Kiendrébéogo, Annick Raissa O. Sidibe, Ghislain Bertrand Compaoré, Relwendé Nacanabo, Orokia Sory, Issa Ouédraogo, Saira Nawaz, Anne E Schuind, Andrew Clark

**Affiliations:** 1 Department of Public Health, University Joseph Ki-Zerbo, Ouagadougou, Burkina Faso; 2 Recherche pour la santé et le développement (RESADE), Ouagadougou, Burkina Faso; 3 Heidelberg Institute of Global Health, Medical Faculty and University Hospital, Heidelberg University, Heidelberg, Germany; 4 Directorate of Prevention through Immunization, Ministry of Health, Ouagadougou, Burkina Faso; 5 Jhpiego, Ouagadougou, Burkina Faso; 6 Radiotherapy Center of Bogodogo, Ouagadougou, Burkina Faso; 7 Institute of Health Sciences and Research (IRSS), Ouagadougou, Burkina Faso; 8 PATH, Seattle, USA; 9 London School of Hygiene and Tropical Medicine, Department of Health Services Research and Policy, London, UK

**Keywords:** HPV vaccine, Economic evaluation, Cost-effectiveness, Modelling study, Burkina Faso

## Abstract

**Background:**

Africa has some of the highest cervical cancer incidence and mortality rates globally. Burkina Faso launched a human papillomavirus (HPV) vaccination programme for 9-year-old girls in 2022 with support from Gavi, the Vaccine Alliance (Gavi). An economic evaluation of HPV vaccination is required to help sustain investment and inform decisions about optimal HPV vaccine choices.

**Methods:**

We used a proportionate outcomes static cohort model to evaluate the potential impact and cost-effectiveness of HPV vaccination for 9-year-old girls over a ten-year period (2022–2031) in Burkina Faso. The primary outcome measure was the cost (2022 US$) per disability-adjusted life year (DALY) averted from a limited societal perspective (including all vaccine costs borne by the government and Gavi, radiation therapy costs borne by the government, and all other direct medical costs borne by patients and their families). We evaluated four vaccines (CERVARIX®, CECOLIN®, GARDASIL-4®, GARDASIL-9®), comparing each to no vaccination (and no change in existing cervical cancer screening and treatment strategies) and to each other. We combined local estimates of HPV type distribution, healthcare costs, vaccine coverage and costs with GLOBOCAN 2020 disease burden data and clinical trial efficacy data. We ran deterministic and probabilistic uncertainty analyses.

**Results:**

HPV vaccination could prevent 37–72% of cervical cancer cases and deaths. CECOLIN® had the most favourable cost-effectiveness (cost per DALY averted < 0.27 times the national gross domestic product [GDP] per capita). When cross-protection was included, CECOLIN® remained the most cost-effective (cost per DALY averted < 0.20 times the national GDP per capita), but CERVARIX® provided greater health benefits (66% vs. 48% reduction in cervical cancer cases and deaths) with similar cost-effectiveness (cost per DALY averted < 0.28 times the national GDP per capita, with CECOLIN® as the comparator). We estimated the annual cost of the vaccination programme at US$ 2.9, 4.1, 4.4 and 19.8 million for CECOLIN®, GARDASIL-4®, CERVARIX® and GARDASIL-9®, respectively. A single dose strategy reduced costs and improved cost-effectiveness by more than half.

**Conclusion:**

HPV vaccination is cost-effective in Burkina Faso from a limited societal perspective. A single dose strategy and/or alternative Gavi-supported HPV vaccines could further improve cost-effectiveness.

**Supplementary Information:**

The online version contains supplementary material available at 10.1186/s12913-023-10283-3.

## Introduction

Cervical cancer is one of the leading causes of cancer death in women, with 604,000 new cases and 342,000 deaths in 2020, worldwide [1, 2]. The countries of sub-Saharan Africa have some of the highest rates of cervical cancer incidence and mortality globally [2]. In Burkina Faso, the annual age-standardized rate of cervical cancer mortality (40 per 100,000 women) is five times higher than the global rate [3, 4] and cervical cancer is the leading cause of cancer mortality in women.

Mainly caused by human papillomavirus (HPV) infection, cervical cancer is preventable through vaccination of pre-adolescent girls [5–8] and effective cervical cancer screening of women above the targeted age of vaccination [9, 10]. Targets set by the World Health Organization (WHO) recommend vaccinating 90% of all girls by age 15 years, screening 70% of women at 35 and 45 years of age, and treating 90% of the precancerous lesions detected by screening programmes [11].

WHO has approved the use of four HPV vaccines [12]: CERVARIX® (GlaxoSmithKline), a bivalent vaccine targeting HPV types 16 and 18; CECOLIN® (Xiamen Innovax Biotech), a bivalent vaccine also targeting HPV types 16 and 18; GARDASIL®, hereafter referred to as GARDASIL-4® (Merck Sharp & Dohme), a quadrivalent vaccine targeting HPV types 6, 11, 16, and 18 (types 6 and 11 are associated with genital warts); [6] and GARDASIL-9® (Merck Sharp & Dohme), a nonavalent vaccine targeting HPV types 6, 11, 16, 18, 31, 33, 45, 52, and 58.

In 2016, the government of Burkina Faso introduced free screening and treatment of precancerous cervical lesions for women aged 25–55 years [13]. From November 2015 to December 2016, the country successfully piloted the use of CERVARIX® in 9-year-old girls [14]. In April 2022, two doses of GARDASIL-4® were introduced for 9-year-old girls as part of the national Expanded Programme on Immunization (EPI) [15]. In 2020, the primary school enrolment rate for girls was 93% [16], and according to the Ministry of Education, more than 87% of 9-year-old girls in Burkina Faso attend school. A combination of school-based and health facility-based delivery was therefore used. A catch-up campaign for girls aged 10–14 years was not adopted.

While there is increasing evidence to demonstrate the cost-effectiveness of HPV vaccination globally, to the best of our knowledge the cost-effectiveness of HPV vaccination has never been estimated in Burkina Faso. This evidence is needed to help sustain investment in the current vaccination programme and help inform decisions about the optimal choice of product in the future. The Ministry of Health (MOH) also needs to consider the potential value of a catch-up campaign, consistent with WHO’s recommendation to protect all girls aged 9–14 years [17]. A single dose strategy has also demonstrated good effectiveness in an African setting and is included by WHO as a recommended option for vaccines where supportive evidence is available [18].

The aim of this study is to estimate the potential health and economic impact of GARDASIL-4® in Burkina Faso and consider alternative policy options that could help to increase the value and/or impact of the current national HPV vaccination programme.

## Methods

### Modelling approach

We used the UNIVAC decision support model (an Excel-based proportionate outcomes static cohort model: www.paho.org/en/provac-toolkit) to evaluate the potential impact and cost-effectiveness of introducing HPV vaccination for 9-year-old girls over a ten year period (2022–2031) in Burkina Faso. UNIVAC is populated with United Nations’ (2019 revision) population estimates of the number of girls alive in each single year and single calendar year of life [19]. Numbers of girls alive in each single year/age of life are multiplied by age-specific rates of cervical cancer cases and deaths to estimate the number of cases, deaths, and disability-adjusted life years (DALYs) expected to occur with and without vaccination over the lifetimes of each cohort of vaccinated girls. The model also estimates the costs of vaccination and the healthcare costs associated with treating cervical cancer cases, with and without vaccination.

The primary outcome measure is the cost (US$) per DALY averted, accounting for all costs and benefits aggregated over the ten cohorts of vaccinated girls (2022–2031). All future costs and health benefits were discounted at 3% per year, and all costs represent 2022 US$ (assuming US $1 = XOF 612.48) [20].

We estimated the potential cost-effectiveness of four different vaccines (CERVARIX®, CECOLIN®, GARDASIL-4®, GARDASIL-9®), comparing each product to no vaccination (and no change in existing cervical cancer screening and treatment strategies) and to each other. In our central estimates, all vaccines were assumed to be administered in two doses without a catch-up campaign. We also estimated the annual undiscounted vaccine programme cost by calendar year to assess the potential budget impact.

Burkina Faso does not have a strict willingness-to-pay (WTP) threshold for determining whether an intervention is cost-effective or not. Ochalek et al. have estimated a WTP threshold for Burkina Faso in the range of 9–29% of the national GDP per capita [21]. We therefore calculated the probability that the vaccine would be cost-effective over a range of alternative possible WTP thresholds between 0 and 1 times the national GDP per capita (US$ 918 in the year 2021) [22].

All model inputs were reviewed during a June 2022 stakeholder consultation workshop. Stakeholders included members of the National Immunization Technical Advisory Group (NITAG), officials from the MOH’s Directorate for Prevention through Immunization, and the research team.

### Disease burden

Inputs used to estimate disease burden are summarised in the Supplementary Table S1. We used age-specific rates of cervical cancer cases and deaths estimated for Burkina Faso by GLOBOCAN for the year 2020 [23]. We used data from Burkina Faso’s national cancer registry to distribute cervical cancer cases into local, regional, and distant stages. Disability weights were taken from the Global Burden of Disease project and represent time lost whilst living with local, regional, and distant cancer [24]. Average five-year survival rates in Burkina Faso were estimated to be 32%, 20%, and 6% for local, regional, and distant cervical cancer, respectively. The five-year survival rate reported for Cote d’Ivoire (23%) [25] was used to rescale stage-specific five-year survival rates from the USA (local = 92%, regional = 58%, distant = 18%, overall = 66%) [26]. For example, the survival rate for local cancer was estimated to be 32% i.e. (23/66) x 92%.

### Healthcare costs

We calculated the cost of cervical cancer treatment, and excluded costs associated with the screening and treatment of precancerous lesions. In Burkina Faso, most cervical cancer treatment costs are paid for out-of-pocket by patients and their families. Radiation therapy, however, is provided free of charge by the government. Our cost perspective therefore included the cost of radiation therapy (borne by the government) and all other direct medical costs borne by patients and their families (e.g., diagnostic costs, assessment of cancer spread, hysterectomy, chemotherapy). We excluded lost wages of patients and their families, and all other costs borne by the government e.g., the health system costs associated with staff, hospital logistics, and facilities.

We estimated the cost of cervical cancer treatment to be US$ 714.30, US$ 923.06, and US$ 985.03 for local, regional, and distant stages, respectively. These costs were calculated from the standard treatment protocols applied in Burkina Faso according to the stage of the cancer as reflected in the Supplementary Figure S1. We used the cancer registry data to distribute patients into local, regional, and distant cancer stages, and sought expert advice (gynaecologists experienced in treating cervical cancer, surgical and medical oncologists, radiotherapists) to assess the treatment regimens used for each stage. The total cost of healthcare at a given stage is the sum of the costs of each diagnosis and treatment procedure performed on the patient. Diagnosis costs included the cost of a biopsy of the uterine cervix and anatomo-pathological examination, pelvic magnetic resonance imaging and gadolinium, thoraco-abdominal CT scan and contrast medium, cystoscopy and/or rectoscopy and anatomical-pathological examination. Treatment costs were stage-specific and included the costs of hysterectomy, radiation therapy, and chemotherapy included the costs of pre-therapy workup (e.g., electrocardiogram, cardiac ultrasound, and blood tests), medical consumables (e.g., IV fluids, gloves, scalpels, infusion sets, intravenous cannula, alcohol, plasters), anticancer drugs, and antiemetics (anti-sickness drugs). The unit costs of each procedure were taken from the tariffs of public hospitals and those of drugs and medical consumables from the standard price list of local pharmacies. We have included the costs of procedures in public hospitals as this is where most patients are treated. These tariffs are displayed or available from hospital billing registers and pharmacies.

### Vaccine programme costs

Input data for vaccine programme costs are summarised in the Supplementary Table S2. Our cost perspective included the full cost of the vaccine borne by the government and Gavi, The Vaccine Alliance (Gavi). The per-dose prices available to Burkina Faso through Gavi are US$ 2.90, US$ 4.60, and US$ 4.50 for CECOLIN®, CERVARIX®, and GARDASIL-4®, respectively. GARDASIL-9® is not yet supported by Gavi, and the cost of self-procurement is unknown. The best negotiated price for a non-Gavi country was US$ 25 per dose, according to the MI4A/V3P vaccine purchase data [27], and we used this price in our model. Prices for other supplies (syringes and safety boxes) were based on data reported in the Burkina Faso immunization forecasting tool provided by UNICEF Supply Division [28].

Handling fees represent the service costs borne by UNICEF as a percentage of the dose price. The fees are established based on total projected annual procurement for a commodity group. We assumed a 3.00% handling fee for all vaccines based on the UNICEF fee applied to new and under-used vaccines in the least-developed countries [29]. We further assumed a 10% international delivery fee to cover the cost of insurance and freight.

We assumed 5% vaccine wastage for vaccines available in a one-dose vial presentation (CECOLIN®, GARDASIL-4®, and GARDASIL-9®) and assumed a two-dose vial for CERVARIX® with 10% wastage.

The incremental health system cost per dose was estimated based on the HPV introduction plan budget made by the government [30]. This was estimated to be US$ 3.50 (range US$ 3.00–4.00) in the first year (2022) and includes communication and demand generation (47% of cost), service delivery (26% of cost), human resource capacity (24% of cost), and program and data management (13% of cost). The recurrent cost per dose in years 2023–2031 was assumed to be US$ 0.91 based on the service delivery fraction (26%).

### Vaccine impact calculations

In our base case scenario, we assumed 85% and 75% vaccine coverage for one and two doses, respectively, in the first year of vaccine introduction; 90% and 80% vaccine coverage for one and two doses, respectively, during the second year; and 100% and 97.5% vaccine coverage for one and two doses, respectively, in the following years, based on the coverage reported in a recent HPV demonstration project [14] and the MOH’s HPV vaccine introduction plan [30]. For each vaccine product, we calculated the percentage distribution of HPV types among cervical cancer cases, applied estimates of vaccine efficacy to each HPV type and summed the products to derive weighted estimates of HPV vaccine efficacy against cervical cancer cases and deaths (see Supplementary Table S3).

**Fig. 1:**
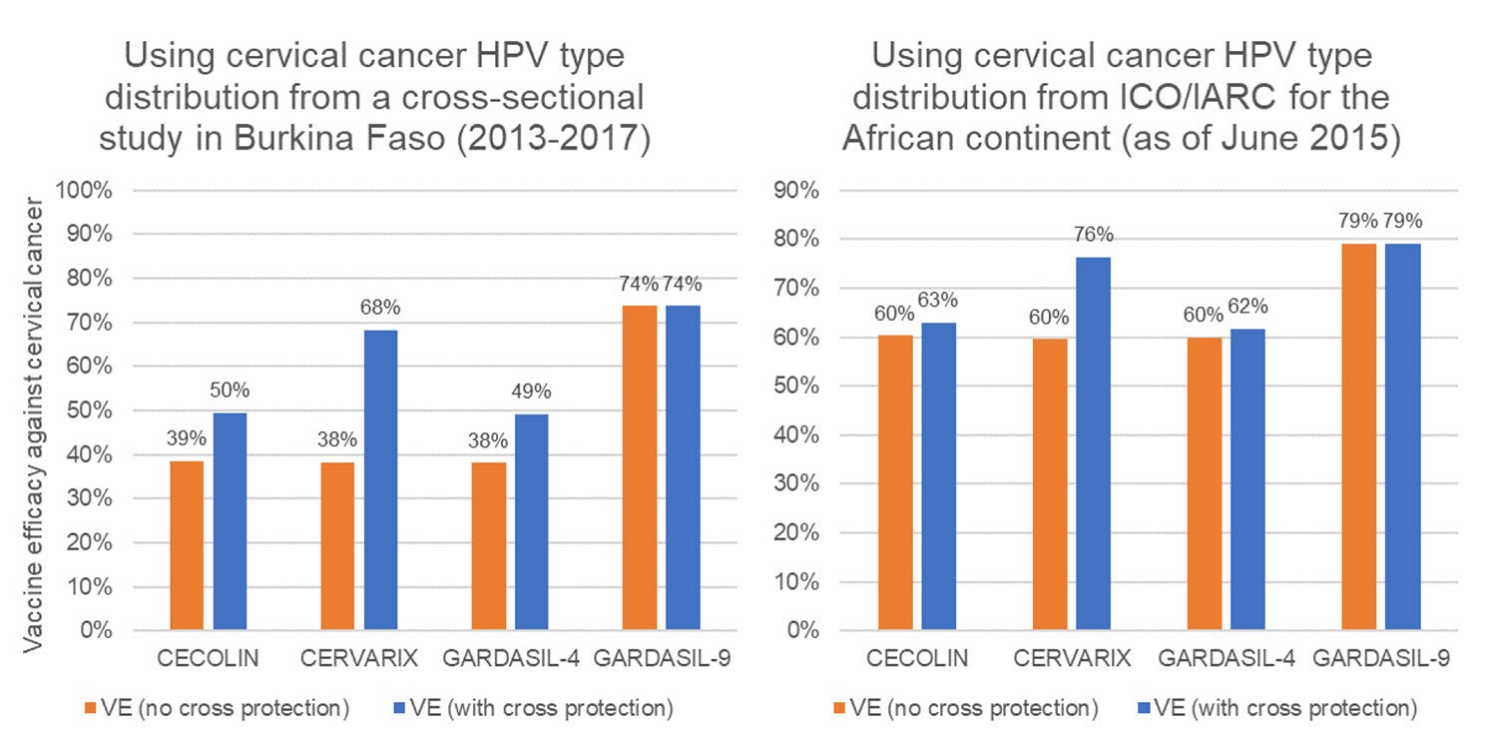
Weighted vaccine efficacy of two doses against cervical cancer cases and deaths by source of HPV type distribution data and type of vaccine product, with and without cross-protection

The HPV type distribution was taken from a cross-sectional multicenter epidemiological study conducted in Burkina Faso between 2013 and 2017 [31]. In this study, HPV genotypes were identified among invasive cervical cancer cases using real-time multiplex PCR. HPV types could only be identified from 72% (47/65) of women with cervical cancer, suggesting a high number of false negatives. We therefore assumed cases without a defined type would have the same HPV type distribution as those with a defined type. The most prevalent HPV types were: 18 (26%), 31 (16%), 16 (13%), 39 (13%), 45 (13%), and 35 (7%). The proportion of cervical cancers caused by vaccine types in this study was much lower than the proportion reported by the Catalan Institute of Oncology/International Agency for Research on Cancer for the African continent [32]. Consequently, our estimates of weighted vaccine efficacy are much lower (and perhaps more conservative) than might be expected for other settings in Africa (Fig. 1).

Estimates of vaccine-type efficacy were taken from Qiao et al. [33] for CECOLIN®, Apter et al. [34] for CERVARIX® and Ault et al. [35] and Garland et al. [36] for GARDASIL-4®. A study by Huh et al. [35] provided additional efficacy data for GARDASIL-9®. There is uncertainty about the scale of cross-protection to non-vaccine types that might be associated with each vaccine product, so we calculated weighted vaccine efficacy with and without cross-protection. For CERVARIX® we assumed there could be cross-protective efficacy against types 31, 33, 45, 51, 52, and 56 based on a study by Wheeler et al. [37]. The authors of this study were uncertain about the benefit associated with types 52 and 56 so we ran a further scenario with the cross-protective effect for types 52 and 56 removed. For GARDASIL-4®, we assumed there could be cross-protection against type 31 based on a study by Brown et al. [38]. We assumed that CECOLIN® would have the same cross-protection as GARDASIL-4®, and no cross-protection was assumed for GARDASIL-9®.

In all scenarios, we assumed one dose of HPV vaccine would provide 80% of the total efficacy assumed for two doses but ran an additional scenario assuming one dose would confer the same level of protection as two doses based on a recent study from Africa and recommendations by WHO [18, 39].

### Uncertainty analysis

We ran simple probabilistic sensitivity analyses (PSA) (1000 runs per scenario) with the mid, low, and high values for each input parameter representing the mode and range within PERT-Beta distributions [40]. Within each run, all parameters other than vaccine price (fixed) were varied across their range. We ran eight PSAs (four vaccines each with and without cross-protection) and presented the distribution of probabilistic run results as clouds on a cost-effectiveness plane. PSA results were also used to inform cost-effectiveness acceptability curves (i.e., the probability that the vaccine would be cost-effective at WTP thresholds ranging from 0 to 1 times the national GDP per capita).

We also ran deterministic sensitivity analyses to show the effect on the cost-effectiveness ratio of changing one input in isolation. The same set of “what-if” scenarios was run for each vaccine with and without cross protection. This included scenarios evaluating a one-dose strategy (we assumed one-dose with full two-dose efficacy and one-dose with two-doses x 0.8 efficacy), a catch-up campaign for ages 10–14 years (we assumed that 95% of girls will receive two doses during catch-up) and a cross-protection scenario for CERVARIX® with the cross-protective effect for types 52 and 56 removed. We also ran a separate scenario assuming the HPV type distribution for Africa rather than using the data from the local study (Fig. 1), a scenario assuming that the cost of cervical cancer treatment was 20% lower and higher than the base case value, and a scenario assuming a higher drop-out rate between the first and second doses, i.e. we halved the dose 2 coverage assumed in the base case scenario.

## Results

Without HPV vaccination, we estimate there could be 62,000 cervical cancer cases and 52,000 cervical cancer deaths over the lifetimes of the ten cohorts of 9-year-old girls (2022–2031). Over the same period, the discounted vaccine programme cost is expected to be US $24.5 million for CECOLIN®, US$ 35 million for GARDASIL-4®, US$ 37 million for CERVARIX®, and US$ 165 million for GARDASIL-9® (Tables 1 and 2).

**Table 1:** Lifetime costs and effects of HPV vaccination in Burkina Faso (2022–2031) assuming no cross-protection

	No vaccine	CECOLIN	GARDASIL-4	CERVARIX	GARDASIL-9
**Lifetime costs and effects**					
Cervical cancer cases (local)	25,684	16,080	16,176	16,138	7,308
Cervical cancer cases (regional)	14,517	9,089	9,143	9,121	4,130
Cervical cancer cases (distant)	21,776	13,633	13,715	13,682	6,196
Cervical cancer cases with treatment	61,976	38,802	39,034	38,941	17,634
Cervical cancer deaths	51,515	32,253	32,446	32,369	14,658
DALYs (discounted*)	221,841	139,162	139,989	139,658	63,640
Vaccine program costs (discounted*)	US$ 0	US$ 24,501,708	US$ 34,663,269	US$ 36,953,148	US$ 164,858,263
Societal healthcare costs (discounted*)	US$ 12,044,932	US$ 7,556,478	US$ 7,601,375	US$ 7,583,416	US$ 3,456,536
**Differences (comparator = no vaccine)**					
Cervical cancer cases (local)	-	9,604	9,508	9,546	18,376
Cervical cancer cases (regional)	-	5,428	5,374	5,396	10,387
Cervical cancer cases (distant)	-	8,143	8,061	8,094	15,580
Cervical cancer cases with treatment	-	23,174	22,942	23,035	44,342
Cervical cancer deaths	-	19,262	19,069	19,146	36,857
DALYs (discounted*)	-	82,679	81,852	82,183	158,201
Vaccine program costs (discounted*)	-	US$ 24,501,708	US$ 34,663,269	US$ 36,953,148	US$ 164,858,263
Societal healthcare costs (discounted*)	-	-US$ 4,488,454	-US$ 4,443,557	-US$ 4,461,516	-US$ 8,588,396
**Cost (US$) per DALY averted (comparator = no vaccine)**					
*Societal cost perspective*	-				
Cost (discounted*)	-	US$ 20,013,254	US$ 30,219,712	US$ 32,509,591	US$ 156,269,867
DALYs averted (discounted*)	-	82,679	81,852	82,183	158,201
Cost per DALY averted (discounted*)	-	US$ 242	Dominated*	Dominated*	US$ 988
Cost (US$) per DALY averted (comparator = next least costly non-dominated** option)					
*Societal cost perspective*	-				
Cost (discounted*)	-	US$ 20,013,254	Dominated**	Dominated**	US$ 136,256,613
DALYs averted (discounted*)	-	82,679	Dominated**	Dominated**	75,522
Cost per DALY averted (discounted*)	-	US$ 242	Dominated**	Dominated**	US$ 1,804

*Future costs/effects were discounted at a rate of 3% per year. ** Dominated options are more expensive and generate fewer benefits than at least one alternative option

**Table 2:** Lifetime costs and effects of HPV vaccination in Burkina Faso (2022–2031) assuming cross-protection

	No vaccine	CECOLIN	GARDASIL-4	CERVARIX	GARDASIL-9
**Lifetime costs and effects**					
Cervical cancer cases (local)	25,684	13,342	13,438	8,698	7,308
Cervical cancer cases (regional)	14,517	7,541	7,595	4,916	4,130
Cervical cancer cases (distant)	21,776	11,312	11,393	7,374	6,196
Cervical cancer cases with treatment	61,976	32,195	32,427	20,989	17,634
Cervical cancer deaths	51,515	26,761	26,954	17,447	14,658
DALYs (discounted*)	221,841	115,589	116,416	75,609	63,640
Vaccine program costs (discounted*)	US$ 0	US$ 24,501,708	US$ 34,663,269	US$ 36,953,148	US$ 164,858,263
Societal healthcare costs (discounted*)	US$ 12,044,932	US$ 6,276,740	US$ 6,321,636	US$ 4,106,332	US$ 3,456,536
**Differences (comparator = no vaccine)**					
Cervical cancer cases (local)	-	12,342	12,246	16,986	18,376
Cervical cancer cases (regional)	-	6,976	6,922	9,601	10,387
Cervical cancer cases (distant)	-	10,464	10,383	14,402	15,580
Cervical cancer cases with treatment	-	29,781	29,549	40,987	44,342
Cervical cancer deaths	-	24,754	24,561	34,068	36,857
DALYs (discounted*)	-	106,252	105,425	146,232	158,201
Vaccine program costs (discounted*)	-	US$ 24,501,708	US$ 34,663,269	US$ 36,953,148	US$ 164,858,263
Societal healthcare costs (discounted*)	-	-US$ 5,768,192	-US$ 5,723,296	-US$ 7,938,600	-US$ 8,588,396
**Cost (US$) per DALY averted (comparator = no vaccine)**					
Societal *cost perspective*	-				
Cost (discounted*)	-	US$ 18,733,516	US$ 28,939,973	US$ 29,014,548	US$ 156,269,867
DALYs averted (discounted*)	-	106,252	105,425	146,232	158,201
Cost per DALY averted (discounted*)	-	US$ 176	US$ 275	US$ 198	US$ 988
Cost (US$) per DALY averted (comparator = next least costly non-dominated** option)					
*Societal cost perspective*	-				
Cost (discounted*)	-	US$ 18,733,516	Dominated**	US$ 10,281,032	US$ 127,255,319
DALYs averted (discounted*)	-	106,252	Dominated**	39,980	11,969
Cost per DALY averted (discounted*)	-	US$ 176	Dominated**	US$ 257	US$ 10,632

*Future costs/effects were discounted at a rate of 3% per year. ** Dominated options are more expensive and generate fewer benefits than at least one alternative option

**Fig. 2:**
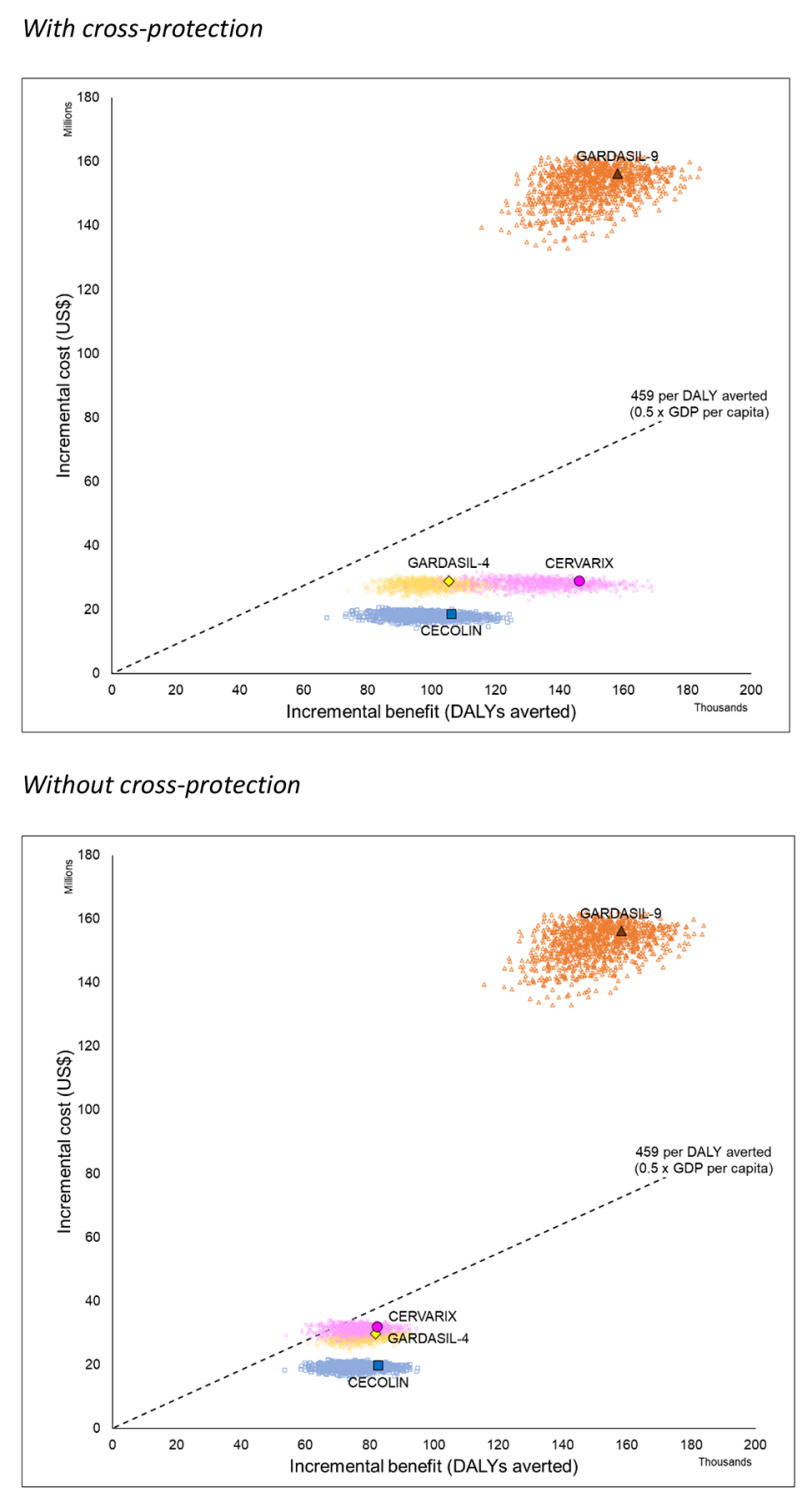
Costs and benefits of alternative HPV vaccines compared to no vaccine and to each other

**Fig. 3:**
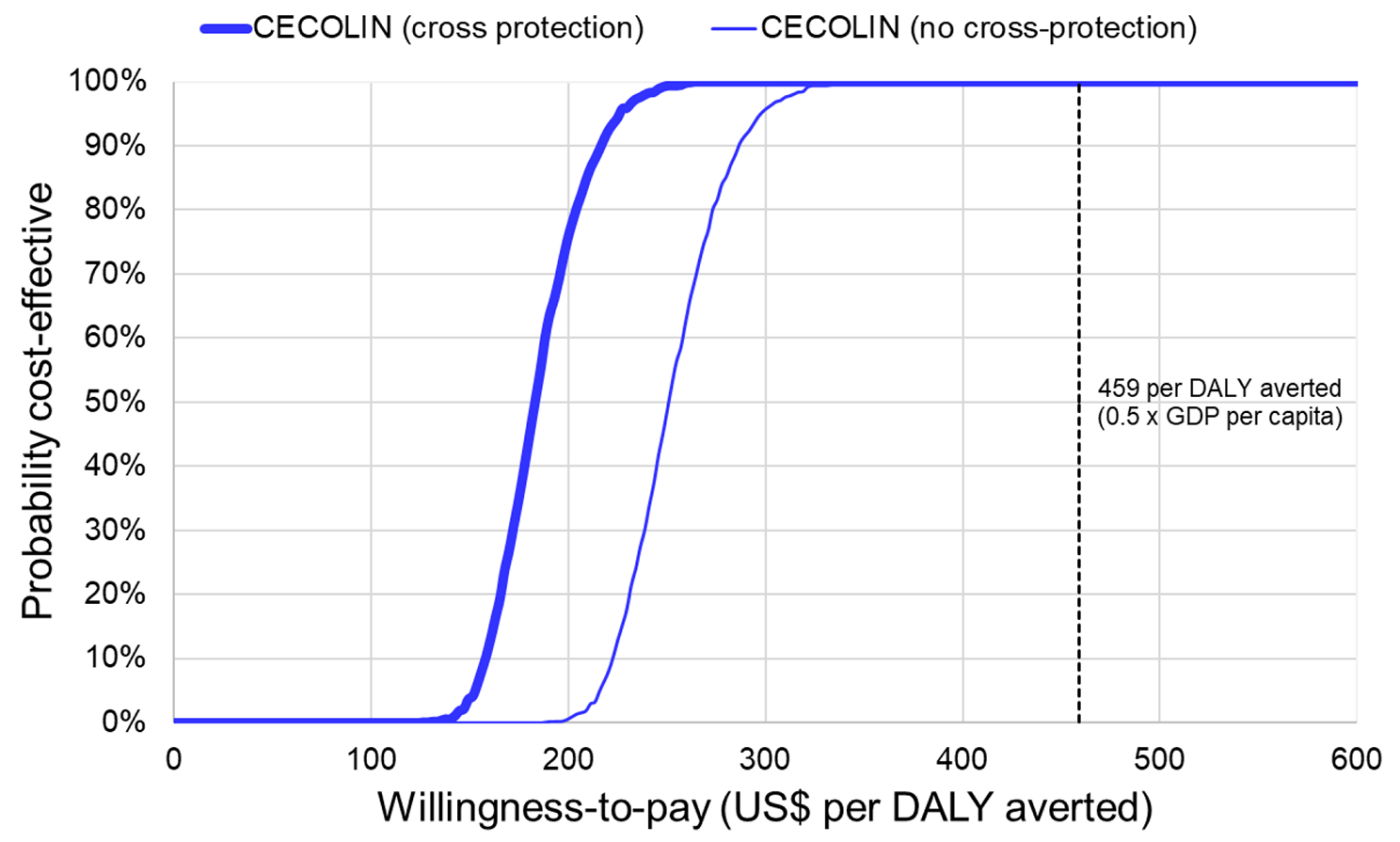
Cost-effectiveness acceptability curve for the vaccine with the most favourable cost-effectiveness (CECOLIN®)

Without cross-protection, the three vaccines supported by Gavi (CECOLIN®, GARDASIL-4®, and CERVARIX®) would be expected to avert 37% of cervical cancer cases and deaths and avert US$ 4.5 million healthcare costs. In contrast, GARDASIL-9® could avert 72% of cervical cancer cases and deaths and avert US$ 8.5 million healthcare costs. CECOLIN® has the lowest estimated net cost (US$ 20 million) and most favourable cost-effectiveness ratio (US$ 242 per DALY averted, or 0.26 times the national GDP per capita in Burkina Faso) (Table 1). CECOLIN® dominates both GARDASIL-4® and CERVARIX® because it is estimated to generate equivalent (slightly higher) benefits at less cost. GARDASIL-9® could achieve more benefit than CECOLIN® but would be substantially more expensive with incremental cost-effectiveness of US$ 1,804 (two times the national GDP per capita) (Table 1; Fig. 2). There is a > 95% probability that the option with the most favourable cost-effectiveness (CECOLIN®) would be cost-effective at a threshold set at around US$ 300 (0.33 times the national GDP per capita) (Fig. 3).

With cross-protection, CECOLIN® and GARDASIL-4® could avert 48% of cervical cancer cases and deaths and avert US$ 5.7 million healthcare costs. In contrast, CERVARIX® could avert 66% of cervical cancer cases and deaths and avert US$ 8 million healthcare costs. Equivalent estimates for GARDASIL-9® were 72% and US$ 8.5 million, respectively. CECOLIN® has the lowest estimated net cost (US$ 18.7 million) and most favourable cost-effectiveness ratio (US$ 176 per DALY averted, or 0.19 times the national GDP per capita in Burkina Faso) (Table 2). CECOLIN® dominates GARDASIL-4® because it generates equivalent (slightly higher) benefits at less cost. CERVARIX® had less favourable net cost than CECOLIN® (US$ 29 million versus US$ 18.7 million) but achieved substantially more health impact (66% versus 48%) and would still have favourable incremental cost-effectiveness (US$ 257 per DALY averted, or 0.28 times the national GDP per capita) when compared directly to CECOLIN®. In contrast, the incremental cost-effectiveness of GARDASIL-9® was very unfavourable when compared directly to CERVARIX® (Table 2; Fig. 2). There is more than 95% probability that the option with the most favourable cost-effectiveness (CECOLIN®) would be cost-effective at a threshold set at around US$ 225 (0.25 times the national GDP per capita) (Fig. 3).

Table 3 shows cost-effectiveness for a range of alternative deterministic “what-if” scenarios. Assuming a single dose strategy had more favourable cost-effectiveness, reducing the cost per DALY averted by more than half. Scenarios using the African HPV type distribution data (rather than the local HPV type distribution) were also influential, improving cost-effectiveness of all three Gavi-supported vaccines by at least one-third: without cross-protection, the cost-effectiveness ratio was 50% lower for CECOLIN®, CERVARIX®, and GARDASIL-4® and 17% lower for GARDASIL-9®; with cross-protection, the ratio was 38%, 25%, and 34% lower for CECOLIN®, CERVARIX®, and GARDASIL-4®, and 17% lower for GARDASIL-9®. However, the rank order and general conclusions of the study remained the same. Assuming a catch-up campaign for ages 10–14 years had less favourable cost-effectiveness—this strategy increased the cost-effectiveness ratio by around 22% for CERVARIX® and GARDASIL-4®, 30% for CECOLIN®, and 10% for GARDASIL-9®.

**Table 3:** Cost (US$) per DALY averted for alternative deterministic scenarios (comparator = no vaccine)

Vaccine	Scenario	Societal perspective	% change	Comment*
**CECOLIN**	**Central inputs (US$ 2.9 per dose for 2 doses with no cross-protection assumption)**	**242**	**-**	Favourable
	With catch-up campaign	312	29%	Favourable
	Schedule = 1 dose with full 2 doses efficacy assumption	102	-58%	Favourable
	Schedule = 1 dose with efficacy = 2 doses efficacy x 0.8 assumption	141	-42%	Favourable
	Vaccine efficacy reported by ICO/IARC for the African continent (www.hpvcentre.net) (1 dose efficacy = 53,76% and 2 doses efficacy = 67,20%)	116	-52%	Favourable
	Cost of cervical cancer treatment 20% lower than the base case value assumption	253	5%	Favourable
	Cost of cervical cancer treatment 20% higher than the base case value assumption	231	-5%	Favourable
	Higher dropout rate between first and second dose assumption. Coverage of second dose assumed in baseline scenario halved	197	-19%	Favourable
	**Central inputs (US$ 2.9 per dose for 2 doses with cross-protection assumption)**	**176**	-	Favourable
	With catch-up campaign	230	31%	Favourable
	Schedule = 1 dose with full 2 doses efficacy assumption	67	-62%	Favourable
	Schedule = 1 dose with efficacy = 2 doses efficacy x 0.8 assumption	98	-44%	Favourable
	Vaccine efficacy reported by ICO/IARC for the African continent (www.hpvcentre.net) (1 dose efficacy = 56,12% and 2 doses efficacy = 70,14%)	109	-38%	Favourable
	Cost of cervical cancer treatment 20% lower than the base case value assumption	187	6%	Favourable
	Cost of cervical cancer treatment 20% higher than the base case value assumption	165	-6%	Favourable
	Higher dropout rate between first and second dose assumption. Coverage of second dose assumed in baseline scenario halved	141	-20%	Favourable
**CERVARIX**	**Central inputs (US$ 4.6 per dose for 2 doses with no cross-protection assumption)**	**396**	**-**	Favourable
	With catch-up campaign	476	20%	Favourable
	Schedule = 1 dose with full 2 doses efficacy assumption	181	-54%	Favourable
	Schedule = 1 dose with efficacy = 2 doses efficacy x 0.8 assumption	239	-40%	Favourable
	Vaccine efficacy reported by ICO/IARC for the African continent (www.hpvcentre.net) (1 dose efficacy = 53,05% and 2 doses efficacy = 66,32%)	206	-48%	Favourable
	Cost of cervical cancer treatment 20% lower than the base case value assumption	406	3%	Favourable
	Cost of cervical cancer treatment 20% higher than the base case value assumption	384	-3%	Favourable
	Higher dropout rate between first and second dose assumption. Coverage of second dose assumed in baseline scenario halved	326	-18%	Favourable
	**Central inputs (US$ 4.6 per dose for 2 doses with cross-protection assumption)**	**198**	-	Favourable
	With catch-up campaign	244	23%	Favourable
	Schedule = 1 dose with full 2 doses efficacy assumption	78	-61%	Favourable
	Schedule = 1 dose with efficacy = 2 doses efficacy x 0.8 assumption	111	-44%	Favourable
	Vaccine efficacy reported by ICO/IARC for the African continent (www.hpvcentre.net) (1 dose efficacy = 68,01% and 2 doses efficacy = 85,01%)	148	-25%	Favourable
	Cost of cervical cancer treatment 20% lower than the base case value assumption	209	6%	Favourable
	Cost of cervical cancer treatment 20% higher than the base case value assumption	188	-5%	Favourable
	Higher dropout rate between first and second dose assumption. Coverage of second dose assumed in baseline scenario halved	159	-20%	Favourable
**GARDASIL-4**	**Central inputs (US$ 4.5 per dose for 2 doses with no cross-protection assumption)**	**369**	**-**	Favourable
	With catch-up campaign	448	21%	Favourable
	Schedule = 1 dose with full 2 doses efficacy assumption	167	-55%	Favourable
	Schedule = 1 dose with efficacy = 2 doses efficacy x 0.8 assumption	223	-40%	Favourable
	Vaccine efficacy reported by ICO/IARC for the African continent (www.hpvcentre.net) (1 dose efficacy = 53,43% and 2 doses efficacy = 66,78%)	188	-49%	Favourable
	Cost of cervical cancer treatment 20% lower than the base case value assumption	380	3%	Favourable
	Cost of cervical cancer treatment 20% higher than the base case value assumption	358	-3%	Favourable
	Higher dropout rate between first and second dose assumption. Coverage of second dose assumed in baseline scenario halved	304	-18%	Favourable
	**Central inputs (US$ 4.5 per dose for 2 doses with cross-protection assumption)**	**275**	-	Favourable
	With catch-up campaign	336	22%	Favourable
	Schedule = 1 dose with full 2 doses efficacy assumption	118	-57%	Favourable
	Schedule = 1 dose with efficacy = 2 doses efficacy x 0.8 assumption	161	-41%	Favourable
	Vaccine efficacy reported by ICO/IARC for the African continent (www.hpvcentre.net) (1 dose efficacy = 54,94% and 2 doses efficacy = 68,67%)	181	-34%	Favourable
	Cost of cervical cancer treatment 20% lower than the base case value assumption	285	4%	Favourable
	Cost of cervical cancer treatment 20% higher than the base case value assumption	264	-4%	Favourable
	Higher dropout rate between first and second dose assumption. Coverage of second dose assumed in baseline scenario halved	224	-19%	Favourable
**GARDASIL-9**	**Central inputs (US$ 25 per dose for 2 doses)**	**988**	**-**	Borderline
	With catch-up campaign	1086	10%	Borderline
	Schedule = 1 dose with full 2 doses efficacy assumption	483	-51%	Favourable
	Schedule = 1 dose with efficacy = 2 doses efficacy x 0.8 assumption	618	-37%	Borderline
	Vaccine price = Highest (US$ 178.14 per dose)	7136	622%	Unfavourable
	Vaccine efficacy reported by ICO/IARC for the African continent (www.hpvcentre.net) (1 dose efficacy = 70,54% and 2 doses efficacy = 88,17%)	818	-17%	Borderline
	Cost of cervical cancer treatment 20% lower than the base case value assumption	999	1%	Borderline
	Cost of cervical cancer treatment 20% higher than the base case value assumption	977	-1%	Borderline
	Higher dropout rate between first and second dose assumption. Coverage of second dose assumed in baseline scenario halved	823	-17%	Borderline

* Favourable, borderline, and unfavourable cost-effectiveness ratios compared to no vaccination

**Fig. 4:**
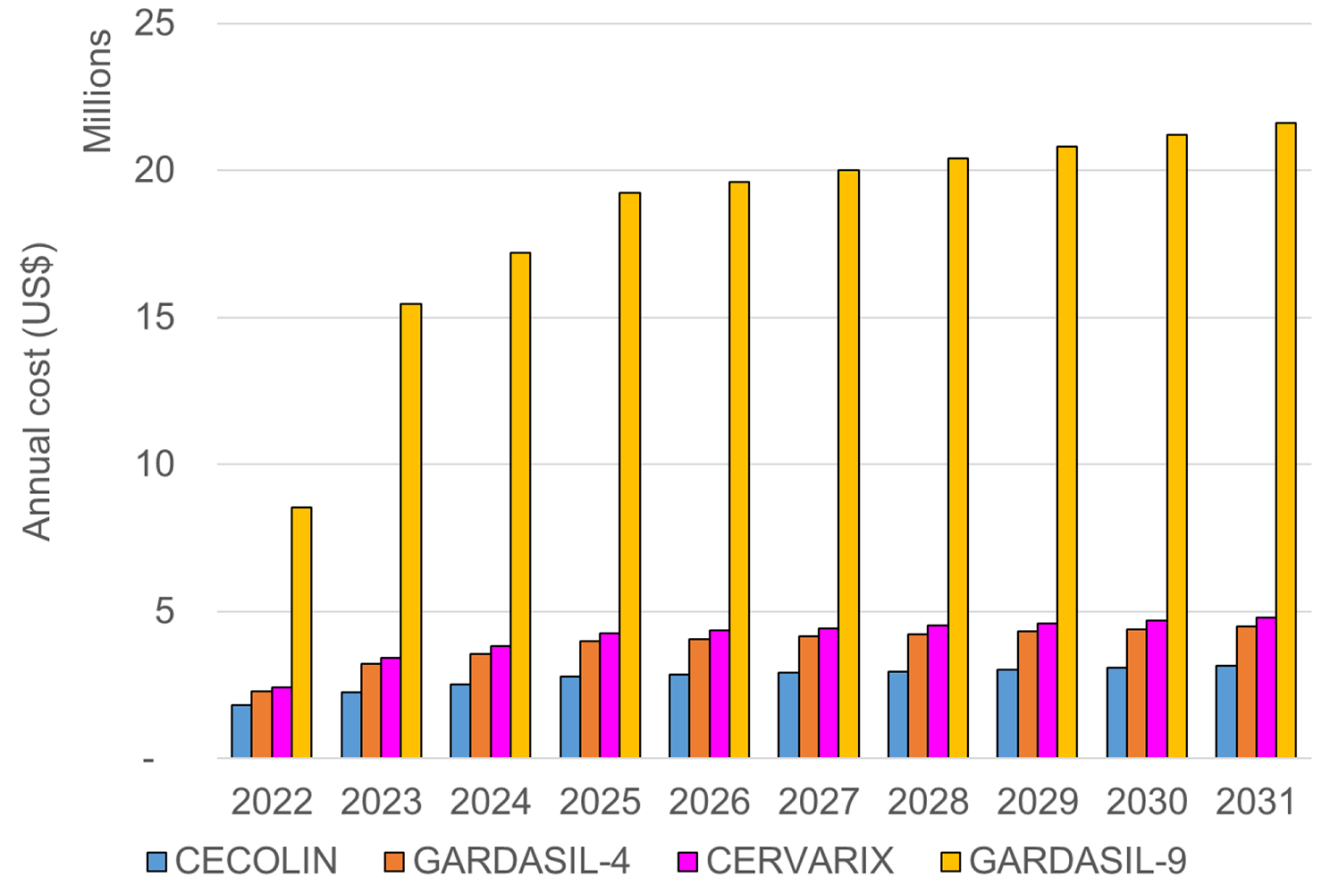
Annual undiscounted vaccine programme cost (US$) of each product compared to no vaccination

Without donor support from Gavi, the median annual undiscounted cost of the vaccination programme was estimated to be US$ 2.9 million for CECOLIN®, US$ 4.1 million for GARDASIL-4®, US$ 4.4 million for CERVARIX®, and US$ 19.8 million for GARDASIL-9® (Fig. 4). The undiscounted total cost of the vaccine programme over the ten years was estimated to be US$ 27.3 million for CECOLIN®, US$ 38.6 million for GARDASIL-4®, US$ 41.2 million for CERVARIX®, and US$ 184 million for GARDASIL-9®.

## Discussion

We estimate that the current HPV vaccination programme in Burkina Faso, using GARDASIL-4®, will prevent a substantial number of cervical cancer cases and deaths. Our assumptions about cross-protection were influential. Without cross-protection, CECOLIN® is likely to be the preferred product, generating lower net costs and similar benefits to both GARDASIL-4® and CERVARIX®. With cross-protection, CECOLIN® also had the most favourable cost-effectiveness, but because CERVARIX® generated substantially more health benefits than CECOLIN® (66% versus 48% vaccine impact), and only slightly less favourable incremental cost-effectiveness (0.28 versus 0.19 times the national GDP per capita), this option should be given serious consideration if affordable. Our findings also suggest that GARDASIL-9® is unlikely to be a viable option unless the current (assumed) price per dose is substantially reduced.

Burkina Faso introduced GARDASIL-4® based on the recommendations of the NITAG after considering the epidemiology of cervical cancer and the availability and affordability of the different products. We assumed a societal perspective including costs borne by both the Government and Gavi. Under this scenario, we find there could be advantages in switching from GARDASIL-4® to either CECOLIN®, or, budget permitting, CERVARIX®. This assumes Burkina Faso will still have access to the vaccine prices assumed in this analysis when it graduates from Gavi support. It is important to emphasise the decision-making perspective considered in our analysis. If we had assumed a narrower payer perspective (excluding any vaccine costs borne by Gavi), then CERVARIX® would have been the most cost-effective option because it was estimated to have the highest health impact. This is because the government co-pay would be the same for any of the three Gavi-supported vaccines. However, irrespective of the decision-maker perspective (with or without costs borne by Gavi) our analysis suggests that switching the current HPV vaccine (GARDASIL-4®) could improve cost-effectiveness. Our analysis did not account for the switching costs involved in replacing GARDASIL-4®, such as health worker training, communication, and monitoring and evaluation. We also did not account for the potential impact of GARDASIL-4® on genital warts, which is one important distinguishing feature of this vaccine. In addition, there is substantial uncertainty around the efficacy and duration of protection of each vaccine product on each HPV type, which makes it difficult to definitively favour one product over another.

In our base case scenario we used HPV type distribution data from a cross-sectional study in Burkina Faso, rather than international database estimates for the African continent. Our cost-effectiveness estimates were substantially more favourable when we re-ran our analysis using the HPV type distribution estimated for the African continent because types 16/18 represented a far higher share of total cervical cancer cases (67% versus 39%). Updated estimates of the HPV type distribution in Burkina Faso would help to clarify the most prominent types in circulation and inform the optimal choice of vaccine product. Our base case assumptions on cross-protection were also uncertain. We included cross-protection against HPV types 52 and 56 but a study by Wheeler et al. [37] has suggested this effect might be due to chance observations. However, both types combined represent < 5% of the total cervical cancer cases in our analysis, so this assumption had a minimal effect on our results.

In our analysis, the vaccine with the most favourable cost-effectiveness (CECOLIN®) reported a cost per DALY averted in the range of 0.19–0.26 times the national GDP per capita in Burkina Faso. Based on historical thresholds of cost-effectiveness (e.g., 1 times national GDP per capita) this would be considered a highly cost-effective intervention. However more stringent and/or context-specific WTP thresholds are now recommended for low- and middle-income countries (LMICs) [41]. For example, Ochalek et al. [21] have proposed a WTP threshold for Burkina Faso in the range of 0.09–0.29 times the national GDP per capita. It is reassuring that CECOLIN® (with or with cross-protection) and CERVARIX® (with cross-protection) could both potentially represent reasonable value for money (< 0.29 times the national GDP per capita) despite our analysis assuming the full cost of the vaccine. In Burkina Faso, households make an important contribution to the healthcare costs associated with cervical cancer, and Gavi make an important contribution to the costs of vaccination. We therefore included both in our estimates of cost-effectiveness. Had we considered a strict ‘Government perspective’ fewer healthcare costs would have been averted (only those associated with radiation therapy), but the cost-effectiveness ratios would have been far more favourable because the Government only pays a small contribution towards the costs of vaccination e.g. $0.20 versus $2.90 per dose of CECOLIN. As the Government will eventually be expected to contribute the full cost of the vaccine, the results for this perspective could be misinterpreted, and the value of HPV vaccination over-stated.

Several studies have found HPV vaccination to be a cost-effective intervention in LMICs [42–44]. A recent meta-regression analysis in 195 countries by Rosettie et al. [43] found that the mean predicted Incremental Cost-Effectiveness Ratio (ICER) for HPV vaccination was US$ 4,217 per DALY averted (95% uncertainty interval [UI]): US$ 773–13,448) worldwide, and sub-Saharan Africa and South Asia had the lowest predicted ICERs, with a population-weighted mean ICER across 46 countries of US$ 706 per DALY averted (95% UI: US$ 130–2,245). However, thresholds based on the national average GDP per capita are likely to mask large sub-national inequalities in the cost and benefits of vaccination. Cost-effectiveness is just one of the decision criteria that should be considered by policy makers. Other criteria include equity, budget impact, feasibility, and acceptability [45].

The cost of the current annual vaccination strategy with GARDASIL-4® ranges from US$ 2.3 million (in 2022) to US$ 4.5 million (in 2031). This annual cost in 2022 represents 31% of the Government’s share of the total EPI budget in 2022, and 3.4% of the total EPI budget (including donor contributions) in 2022. In Ghana, the projected average annual undiscounted costs of the HPV vaccine programme represented 11–15% of the total immunization costs for 2022 [46]. We find that a single dose strategy could further reduce costs and improve cost-effectiveness. WHO’s Strategic Advisory Group of Experts on Immunization recently recommended the use of either one or two doses of CERVARIX®, GARDASIL-4®, and GARDASIL-9® [47]. The option to use a single dose is based on available evidence and therefore may not apply to CECOLIN® at this time.

Burkina Faso launched a nationwide HPV vaccination programme for 9-year-old girls in May 2022 without a catch-up campaign for girls aged 10–14 years. Scenarios with a catch-up campaign had a higher cost per DALY averted, but the cost-effectiveness ratios were still relatively favourable. This strategy should therefore be considered to protect girls currently aged 10–14 years who were not eligible for routine HPV vaccination at age 9 years. If costs are prohibitive, then a single dose strategy could be considered to reduce the cost. A further enhancement to the programme would be to include HPV vaccination for boys, but evaluation of this would require the use of a more complicated model, and was therefore outside the scope of our current study.

Our analysis was conservative (unfavourable to HPV vaccination) for several reasons. First, we assumed the full cost of the vaccines rather than simply assuming the proportion contributed by the government in the initial phase of Gavi support. Second, we assumed a limited societal perspective, excluding lost wages of patients and their families, and any health system healthcare costs borne by the government. Third, we probably underestimated the burden of cervical cancer because some sick women will not be included in official statistics. Fourth, we used a simple static cohort model, and have therefore not captured any additional indirect (herd immunity) benefits associated with vaccination. In contrast, our analysis may have favoured HPV vaccination by assuming relatively low incremental health system costs despite very high vaccine programme coverage for two doses (97.5% from year 3). Despite more than 87% of 9-year-old girls attending school in Burkina Faso [16], this optimistic level of programme coverage may still require higher incremental health system costs per dose than we have assumed in our analysis. Our estimates of the incremental health system costs also have excluded the cost of activities that were covered by a Gavi vaccine introduction grant. This was estimated to be US $1,033,385 in the first year of introduction and represents US$ 3.15 per vaccinated child, distributed between Gavi support (US$ 2.4 per vaccinated child), other partners (US$ 0.34 per vaccinated child), and the government (US$ 0.41 per vaccinated child) [30]. However, due to delays, programmatic challenges and exchange rate fluctuations, the governmental cost contribution was higher than planned. Finally, we assumed the counterfactual (without vaccination) rate of cervical cancer cases and deaths would remain constant with time.

## Conclusion

HPV vaccination is estimated to prevent a substantial number of cervical cancer cases and deaths in Burkina Faso. HPV vaccination is cost-effective in Burkina Faso from a limited societal perspective. A single dose strategy and/or alternative Gavi-supported HPV vaccines should be considered to further improve cost-effectiveness.

### Electronic supplementary material

Below is the link to the electronic supplementary material.

Supplementary Material 1

Supplementary Material 2

Supplementary Material 3

Supplementary Material 4

## Data Availability

All data generated or analysed during this study are included in this published article [and its supplementary information files containing web links].

## List of abbreviations

DALY: disability-adjusted life year
EPI: Expanded Programme on Immunization
Gavi: Gavi, the Vaccine Alliance
GDP: gross domestic product
HPV: human papillomavirus
ICER: Incremental Cost-Effectiveness Ratio
LMICs: low- and middle-income countries
MOH: Ministry of Health
NITAG: National Immunization Technical Advisory Group
PSA: probabilistic sensitivity analyses
WTP: willingness-to-pay
WHO: World Health Organization
