# Supplementary figures and images for: Cost-effectiveness of human papillomavirus (HPV) vaccination in Burkina Faso: a modelling study
Source: BMC Health Serv Res. 2023 Dec 1;23:1338. doi: 10.1186/s12913-023-10283-3 (PMC10693094; doi:10.1186/s12913-023-10283-3)

**Supplementary Figure S1. Distribution of cervical cancer cases by stage and treatment pathway**


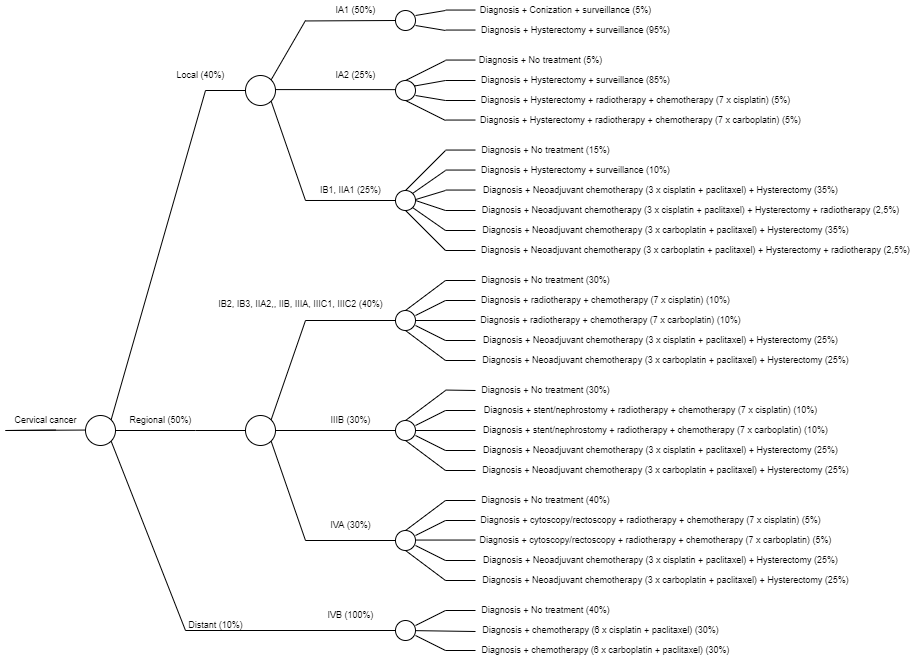

Supplement: Supplementary file 1 — Supplementary Material 1 [file 12913_2023_10283_MOESM1_ESM.docx]
