# Supplementary material for: Cost-effectiveness of human papillomavirus (HPV) vaccination in Burkina Faso: a modelling study
Source: BMC Health Serv Res. 2023 Dec 1;23:1338. doi: 10.1186/s12913-023-10283-3 (PMC10693094; doi:10.1186/s12913-023-10283-3)
Supplement: Supplementary file 2 — Supplementary Material 2 [file 12913_2023_10283_MOESM2_ESM.docx]

**Supplementary Table S1. Input parameters for estimating cervical cancer disease burden**

| **Parameter** | **Value** | **Low** | **High** | **Source/s** |
| --- | --- | --- | --- | --- |
| **Annual rate of cervical cancer DEATHS per 100,000 females** | | | | |
| 10–14yrs | 0.0 | 0.0 | 0.0 | Globocan 2020 -/+20% (https://gco.iarc.fr/today/) |
| 15–19yrs | 0.0 | 0.0 | 0.0 | Globocan 2020 -/+20% (https://gco.iarc.fr/today/) |
| 20–24yrs | 0.0 | 0.0 | 0.0 | Globocan 2020 -/+20% (https://gco.iarc.fr/today/) |
| 25–29yrs | 2.3 | 1.8 | 2.8 | Globocan 2020 -/+20% (https://gco.iarc.fr/today/) |
| 30–34yrs | 6.7 | 5.4 | 8.0 | Globocan 2020 -/+20% (https://gco.iarc.fr/today/) |
| 35–39yrs | 12.8 | 10.2 | 15.4 | Globocan 2020 -/+20% (https://gco.iarc.fr/today/) |
| 40–44yrs | 21.1 | 16.9 | 25.3 | Globocan 2020 -/+20% (https://gco.iarc.fr/today/) |
| 45–49yrs | 30.6 | 24.5 | 36.7 | Globocan 2020 -/+20% (https://gco.iarc.fr/today/) |
| 50–54yrs | 41.9 | 33.5 | 50.3 | Globocan 2020 -/+20% (https://gco.iarc.fr/today/) |
| 55–59yrs | 52.0 | 41.6 | 62.4 | Globocan 2020 -/+20% (https://gco.iarc.fr/today/) |
| 60–64yrs | 58.6 | 46.9 | 70.3 | Globocan 2020 -/+20% (https://gco.iarc.fr/today/) |
| 65–69yrs | 58.3 | 46.6 | 70.0 | Globocan 2020 -/+20% (https://gco.iarc.fr/today/) |
| 70–74yrs | 52.6 | 42.1 | 63.1 | Globocan 2020 -/+20% (https://gco.iarc.fr/today/) |
| 75–79yrs | 39.3 | 31.4 | 47.2 | Globocan 2020 -/+20% (https://gco.iarc.fr/today/) |
| 80–84yrs | 21.8 | 17.4 | 26.2 | Globocan 2020 -/+20% (https://gco.iarc.fr/today/) |
| 85–89yrs | 0.0 | 0.0 | 0.0 | Globocan 2020 -/+20% (https://gco.iarc.fr/today/) |
| 90–94yrs | 0.0 | 0.0 | 0.0 | Globocan 2020 -/+20% (https://gco.iarc.fr/today/) |
| 95–99yrs | 0.0 | 0.0 | 0.0 | Globocan 2020 -/+20% (https://gco.iarc.fr/today/) |
| **Annual incidence of cervical cancer CASES per 100,000 females** | | | |  |
| 10–14yrs | 0.0 | 0.0 | 0.0 | Globocan 2020 -/+20% (https://gco.iarc.fr/today/) |
| 15–19yrs | 0.0 | 0.0 | 0.0 | Globocan 2020 -/+20% (https://gco.iarc.fr/today/) |
| 20–24yrs | 0.0 | 0.0 | 0.0 | Globocan 2020 -/+20% (https://gco.iarc.fr/today/) |
| 25–29yrs | 3.7 | 3.0 | 4.4 | Globocan 2020 -/+20% (https://gco.iarc.fr/today/) |
| 30–34yrs | 14.0 | 11.2 | 16.8 | Globocan 2020 -/+20% (https://gco.iarc.fr/today/) |
| 35–39yrs | 24.4 | 19.5 | 29.3 | Globocan 2020 -/+20% (https://gco.iarc.fr/today/) |
| 40–44yrs | 35.4 | 28.3 | 42.5 | Globocan 2020 -/+20% (https://gco.iarc.fr/today/) |
| 45–49yrs | 45.6 | 36.5 | 54.7 | Globocan 2020 -/+20% (https://gco.iarc.fr/today/) |
| 50–54yrs | 50.7 | 40.6 | 60.8 | Globocan 2020 -/+20% (https://gco.iarc.fr/today/) |
| 55–59yrs | 58.1 | 46.5 | 69.7 | Globocan 2020 -/+20% (https://gco.iarc.fr/today/) |
| 60–64yrs | 62.0 | 49.6 | 74.4 | Globocan 2020 -/+20% (https://gco.iarc.fr/today/) |
| 65–69yrs | 61.3 | 49.0 | 73.6 | Globocan 2020 -/+20% (https://gco.iarc.fr/today/) |
| 70–74yrs | 53.7 | 43.0 | 64.4 | Globocan 2020 -/+20% (https://gco.iarc.fr/today/) |
| 75–79yrs | 39.3 | 31.4 | 47.2 | Globocan 2020 -/+20% (https://gco.iarc.fr/today/) |
| 80–84yrs | 17.4 | 13.9 | 20.9 | Globocan 2020 -/+20% (https://gco.iarc.fr/today/) |
| 85–89yrs | 0.0 | 0.0 | 0.0 | Globocan 2020 -/+20% (https://gco.iarc.fr/today/) |
| 90–94yrs | 0.0 | 0.0 | 0.0 | Globocan 2020 -/+20% (https://gco.iarc.fr/today/) |
| 95–99yrs | 0.0 | 0.0 | 0.0 | Globocan 2020 -/+20% (https://gco.iarc.fr/today/) |
| **% distribution of cervical cancer by severity*** | | | | |
| % Local cancer (n) | 41.4 (46) | - | - | National Cancer Registry |
| % Regional cancer (n) | 23.4 (26) | - | - | National Cancer Registry |
| % Distant cancer (n) | 35.2 (39) | - | - | National Cancer Registry |
| **Disability weights for DALYs calculations** | |  |  |  |
| % of time lost (local cancer) | 28.8 | 19.3 | 39.9 | Salomon J. 2015 (proxy: Diagnosis and primary therapy)^24^ |
| % of time lost (regional cancer) | 45.1 | 30.7 | 60.0 | Salomon J. 2015 (proxy: Metastatic phase)^24^ |
| % of time lost (distant cancer) | 54.0 | 37.7 | 68.7 | Salomon J. 2015 (proxy: Terminal phase)^24^ |
| **5-year survival rate (% alive after 5 years)** | | |  |  |
| Local cancer | 32 | 24 | 40 | Cote d’Ivoire^25^ and USA^26^ -/+25% |
| Regional cancer | 20 | 15 | 25 | Cote d’Ivoire^25^ and USA^26^ -/+25% |
| Distant cancer | 6 | 4.5 | 7.5 | Cote d’Ivoire^25^ and USA^26^ -/+25% |
|  |  |  |  |  |
| ^*^ Figure 1 describes how cancer cases were distributed into local, regional, and distant cancer. | | | | |
