# Supplementary material for: Cost-effectiveness of human papillomavirus (HPV) vaccination in Burkina Faso: a modelling study
Source: BMC Health Serv Res. 2023 Dec 1;23:1338. doi: 10.1186/s12913-023-10283-3 (PMC10693094; doi:10.1186/s12913-023-10283-3)
Supplement: Supplementary file 3 — Supplementary Material 3 [file 12913_2023_10283_MOESM3_ESM.docx]

**Supplementary Table S2. Input parameters for estimating HPV vaccine programme costs**

| **Parameter** | **Value** | **Low** | **High** | **Source/s** | |
| --- | --- | --- | --- | --- | --- |
| **Price of vaccine doses** |  |  |  | |  |
| CECOLIN | US$ 2.90 | - | - | | [HPV-vaccine-prices.pdf (unicef.org)](https://www.unicef.org/supply/media/11776/file/HPV-vaccine-prices.pdf) |
| CERVARIX | US$ 4.60 | - | - | | [HPV-vaccine-prices.pdf (unicef.org)](https://www.unicef.org/supply/media/11776/file/HPV-vaccine-prices.pdf) |
| GARDASIL-4 | US$ 4.50 | - | - | | [HPV-vaccine-prices.pdf (unicef.org)](https://www.unicef.org/supply/media/11776/file/HPV-vaccine-prices.pdf) |
| GARDASIL-9 | US$ 25.00 | - | - | | MI4A/V3P Vaccine Purchase Database (WHO) |
| **International handling (% of price)** |  |  |  | |  |
| CECOLIN | 3% | - | - | | <https://www.unicef.org/supply/handling-fees> |
| CERVARIX | 3% | - | - | | <https://www.unicef.org/supply/handling-fees> |
| GARDASIL-4 | 3% | - | - | | <https://www.unicef.org/supply/handling-fees> |
| GARDASIL-9 | 3% | - | - | | <https://www.unicef.org/supply/handling-fees> |
| **International delivery (% of price)** |  |  |  | |  |
| CECOLIN | 10% | 8% | 12% | | Assumption -/+20% |
| CERVARIX | 10% | 8% | 12% | | Assumption -/+20% |
| GARDASIL-4 | 10% | 8% | 12% | | Assumption -/+20% |
| GARDASIL-9 | 10% | 8% | 12% | | Assumption -/+20% |
| **Wastage percentage** |  |  |  | |  |
| CECOLIN | 5% | 4% | 6% | | <https://www.gavi.org/sites/default/files/support/Gavi_HPV_vaccine_profiles.pdf> -/+20% |
| CERVARIX | 10% | 8% | 12% | |  |
| GARDASIL-4 | 5% | 4% | 6% | |  |
| GARDASIL-9 | 5% | 4% | 6% | |  |
| **Costs of syringes** |  |  |  | |  |
| Price per dose: | US$ 0.08 | - | - | | Burkina Faso Immunization Forecasting Tool |
| % international handling: | 3% | - | - | | <https://www.unicef.org/supply/handling-fees> |
| % international delivery: | 10% | 8% | 12% | | Assumption -/+20% |
| % wastage ^*^ | 5% | 4% | 6% | | Assumption -/+20% |
| **Costs of safety box** |  |  |  | |  |
| Price per syringe/dose: | US$ 0.01 | - | - | | Burkina Faso Immunization Forecasting Tool |
| % international handling: | 3% | - | - | | <https://www.unicef.org/supply/handling-fees> |
| % international delivery: | 10% | 8% | 12% | | Assumption -/+20% |
| % wastage ^*^ | 5% | 4% | 6% | | Assumption -/+20% |
| **Incremental health system costs** |  |  |  | |  |
| Cost per dose (year 1) | US$ 3.50 | US$ 3.00 | US$ 4.00 | | HPV operational budget^30^ |
| Cost per dose (years 2–10) | US$ 0.91 | US$ 0.50 | US$ 1.00 | | HPV operational budget^30^ |
|  |  |  |  | |  |
| ^*^ The % wastage is converted into a factor [1/ (1 - % wastage)] which is multiplied by the expected number of doses required to meet the anticipated level of coverage. | | | | | |
