# Supplementary material for: Cost-effectiveness of human papillomavirus (HPV) vaccination in Burkina Faso: a modelling study
Source: BMC Health Serv Res. 2023 Dec 1;23:1338. doi: 10.1186/s12913-023-10283-3 (PMC10693094; doi:10.1186/s12913-023-10283-3)
Supplement: Supplementary file 4 — Supplementary Material 4 [file 12913_2023_10283_MOESM4_ESM.docx]

**Supplementary Table S3. Input parameters for estimating the impact of HPV vaccination on cervical cancer cases and deaths**

| **Parameter** | **Value** | **Low** | **High** | **Source/s** |
| --- | --- | --- | --- | --- |
| **Vaccine programme coverage** | | | | |
| 1 dose | 85% (2022)  90% (2023)  100% (2024–2031) | 68%  72%  80% | 100%  100%  100% | HPV demonstration report, Burkina Faso’s MOH, 2015–2017^14^ |
| 2 doses | 75% (2022)  80% (2023)  97.5% (2024–2031) | 60%  64%  78% | 90%  96%  100% | Projection. HPV vaccine introduction plan, Burkina Faso’s MOH^30^ |
| **Vaccine efficacy against all types (with cross-protection)** | | | | |
| CECOLIN |  |  |  |  |
| 1 dose | 39.65% | 25.51% | 41.94% | Assumption (80% of two doses VE) |
| 2 doses | 49.57% | 31.89% | 52.43% | Qiao JNCI 2019^33^ |
| CERVARIX |  |  |  |  |
| 1 dose | 54.57% | 35.13% | 56.33% | Assumption (80% of two doses VE) |
| 2 doses | 68.22% | 43.92% | 70.41% | Apter 2015,^34^ Wheeler 2012^37^ |
| GARDASIL-4 |  |  |  |  |
| 1 dose | 39.34% | 30.67% | 41.84% | Assumption (80% of two doses VE) |
| 2 doses | 49.18% | 38.34% | 52.30% | Ault 2007^35^, Brown 2009^38^ |
| GARDASIL-9 |  |  |  |  |
| 1 dose | 59.04% | 49.73% | 60.41% | Assumption (80% of two doses VE) |
| 2 doses | 73.80% | 62.16% | 75.51% | Ault 2007 ^35^ , Huh 2017 (suppl)^48^ |
| **Vaccine efficacy against vaccine types (no cross-protection)** | | | | |
| CECOLIN |  |  |  |  |
| 1 dose | 30.86% | 21.47% | 30.86% | Assumption (80% of two doses VE) |
| 2 doses | 38.57% | 26.84% | 38.57% | Qiao JNCI 2019^33^ |
| CERVARIX |  |  |  |  |
| 1 dose | 30.67% | 21.77% | 30.86% | Assumption (80% of two doses VE) |
| 2 doses | 38.34% | 27.22% | 38.57% | Apter 2015^34^ |
| GARDASIL-4 |  |  |  |  |
| 1 dose | 30.55% | 26.64% | 30.75% | Assumption (80% of two doses VE) |
| 2 doses | 38.18% | 33.30% | 38.44% | Ault 2007^35^ / Garland 2007^36^ |
| GARDASIL-9 |  |  |  |  |
| 1 dose | 59.04% | 49.73% | 60.41% | Assumption (80% of two doses VE) |
| 2 doses | 73.80% | 62.16% | 75.51% | Ault 2007 ^35^, Huh 2017^48^, Garland 2007^36^ |

HPV, human papillomavirus; MOH, Ministry of Health; VE, vaccine effectiveness.

We assumed the type of distribution from Zohoncon.^31^ Cross protective efficacy was assumed against HPV types 31, 33, 45, 51, 52 and 56 for CERVARIX,^37^ and against type 31 for GARDASIL-4.^38^ We further assumed the same cross-protection against type 31 for CECOLIN.
